# Correlation of Coding and Non-Coding RNAs on the Fat Deposition of Yaks Under Different Feeding Systems

**DOI:** 10.3390/ijms26115359

**Published:** 2025-06-03

**Authors:** Lin Xiong, Jie Pei, Shaoke Guo, Mengli Cao, Zhiqiang Ding, Yandong Kang, Xiaoyun Wu, Xian Guo

**Affiliations:** 1 Key Laboratory of Yak Breeding in Gansu Province, Lanzhou Institute of Husbandry and Pharmaceutical Sciences, Chinese Academy of Agricultural Sciences, Lanzhou 730050, China; xionglin@caas.cn (L.X.); peijie@caas.cn (J.P.); gsk1125@163.com (S.G.); caomengliaaa@163.com (M.C.); dingziqiang1997@163.com (Z.D.); kangyandong0901@163.com (Y.K.); wuxiaoyun@caas.cn (X.W.); 2 Key Laboratory of Animal Genetics and Breeding on Tibetan Plateau, Ministry of Agriculture and Rural Affairs, Lanzhou 730050, China

**Keywords:** yak, fat deposition, regulatory gene, non-coding RNAs, signal pathway

## Abstract

The yak is a classic grazing livestock species on the Qinghai–Tibet Plateau, and fat deposition is indispensable for its survival and metabolism. Coding and non-coding RNAs (ncRNAs) play an important role in regulating fat deposition in livestock. In this study, the expression of mRNAs, lncRNAs, miRNAs, and circRNAs in the subcutaneous fat of yaks under grazing and stall feeding was measured using whole-transcriptome sequencing technology. A total of 677 differentially expressed (DE) mRNAs, 120 DE lncRNAs, 2216 DE circRNAs, and 15 DE miRNAs were identified, and their biological function was explored using Gene Ontology (GO) and Kyoto Encyclopedia of Genes and Genomes (KEGG) analyses. Co-expression RNA (ceRNA) networks between DE ncRNAs and DE mRNAs were further constructed, and the crucial RNAs and signal pathways regulating fat deposition in yaks were obtained. The effect of mRNAs and ncRNAs on fat deposition in yaks mainly depended on the PPAR, PI3K–Akt, and cAMP signaling pathways, and the regulatory pathways *TCONS00042948*, *TCONS00012083/bta-miR-2316/MCAT*, and *NR4A3* may be critical in this process. This study provides some theoretical basis for breeding yak species and promotes improvements in yak production.

## 1. Introduction

The yak (*Bos grunniens*) mainly lives on the Qinghai–Tibet Plateau and its adjacent areas and provides animal-derived food, power, and fuel to local people [1]. Sufficient fat deposition can support the yak’s normal metabolism under the harsh natural conditions of the cold season; it is also essential for normal pregnancy and lactation in female yaks [2]. Insufficient fat in yaks usually results in death during the cold season. Moreover, fat characteristics greatly affect yak meat quality and processability. The tenderness, succulency, and color of yak meat are poor in quality compared to cattle, which is largely due to the lower content of intramuscular fat. Fat deposition in livestock is affected by multiple factors, including genetics, age, diet, environment, feeding system, and gender [3,4,5]. Overgrazing on the Qinghai–Tibet Plateau has led to serious grassland degradation over the last few decades, which has limited the development of traditional yak production. An increasing number of herdsmen and farming enterprises recognize that the sustainable development of yak production can only be realized through a transition from traditional grazing (GF). Stall feeding (SF) can significantly increase yak growth performance, and our previous study showed that the capacity of fat deposition in yaks under SF was significantly higher than that of yaks under GF [6]. The expression of specific genes in time and space regulates fat deposition in livestock from the angle of molecular biology, and the feeding system also indirectly affects the expression of genes regulating fat metabolism. The effect of mRNAs on yak fat deposition has been reported [7], but there are very few correlational studies of mRNAs and non-coding RNAs (ncRNAs) in yaks from the perspective of whole-transcriptome sequencing.

There are complex regulatory relationships between mRNAs and ncRNAs, and exploring their potential interactions has become a new trend in transcriptomics research [8,9]. Full-transcriptome sequencing technology simultaneously studies the expression of mRNAs and ncRNAs in specific tissues and has been used to reveal transcriptional regulation among all RNAs [10]. Many mRNAs, lncRNAs, miRNAs, and circRNAs are closely related to adipogenesis [11]. CircRNAs regulate triglyceride accumulation by influencing the PPAR and AMPK signaling pathways [12]; the target genes of lncRNAs are associated with adipocyte differentiation [13]; and miRNAs regulate adipocyte proliferation and differentiation and fat metabolism. Some mRNAs, lncRNAs, miRNAs, and circRNAs that regulate fat deposition in pigs, chickens, goats, and bovines have been found using full-transcriptome sequencing technology [14]. Relevant studies on bovines mainly focus on screening crucial ncRNAs regulating fat deposition, and some signaling pathways have also been found and verified. MiRNAs are closely related to adipocyte proliferation and differentiation in cattle [15]. LncRNA *ADNCR*, as an *miR-204* sponge, suppresses the lipogenesis in bovine preadipocytes by regulating the expression of the *SIRT1* gene [16]; *circFUT10*, via sponging *let-7c*, relieves the inhibition of *let-7c* to the *PPARGC1B* gene, which promotes the proliferation of bovine adipocytes and inhibits cell differentiation [17]. However, there are very few reports on lncRNAs, miRNAs, and circRNAs regulating yak fat deposition, as far as we know.

In this study, the expression of all RNAs in yak subcutaneous fat under GF and SF was detected using whole-transcriptome sequencing technology. Differentially expressed mRNAs (DE mRNAs), lncRNAs (DE lncRNAs), circRNAs (DE circRNAs), and miRNAs (DE miRNAs) were selected, and their biological functions in regulating fat deposition in yaks were explored using Gene Ontology (GO) and Kyoto Encyclopedia of Genes and Genomes (KEGG) analyses. Co-expression RNA (ceRNA) networks among the DE RNAs were further constructed, and the crucial RNAs and regulatory pathways involved in yak fat deposition were explored. This study provides some new insights into yak fat deposition through the interactions between ncRNAs and mRNAs and establishes a new theoretical basis for comprehensively understanding its regulatory pathways.

## 2. Results

### 2.1. Overview of Sequencing Data from Yak Subcutaneous Fat

A total of 528.93 M raw reads, 79.34 G raw bases, 524.14 M clean reads, and 77.25 G clean bases were obtained from all yak fat samples. The preprocessing results of sequencing data quality are shown in Table S1. The clean bases ranged from 12.01 to 14.04 G, and the percentage of valid bases ranged from 97.05% to 97.62%. The Q30 value ranged from 93.94% to 94.06%, while the GC content ranged from 50.71% to 52.30%. Furthermore, 92.72–93.3% of clean reads were successfully mapped to the yak genome. Among these successfully mapped reads, 78.92% of uniquely mapped reads were used for transcript construction. For small RNA sequencing, a total of 150.11 M raw reads, 145.66 M reads with trimmed length, 144.65 M reads with trimmed Q20, 144.53 M reads with trimmed N, and 144.53 M clean reads were obtained. The clean reads ranged from 23.99 to 24.21 M. The genome comparison rate ranged from 95.54% to 96.36%, and the comparison rate of known miRNAs ranged from 72.51% to 76.98%.

### 2.2. Properties of the RNAs in Yak Subcutaneous Fat

**Figure 1:**
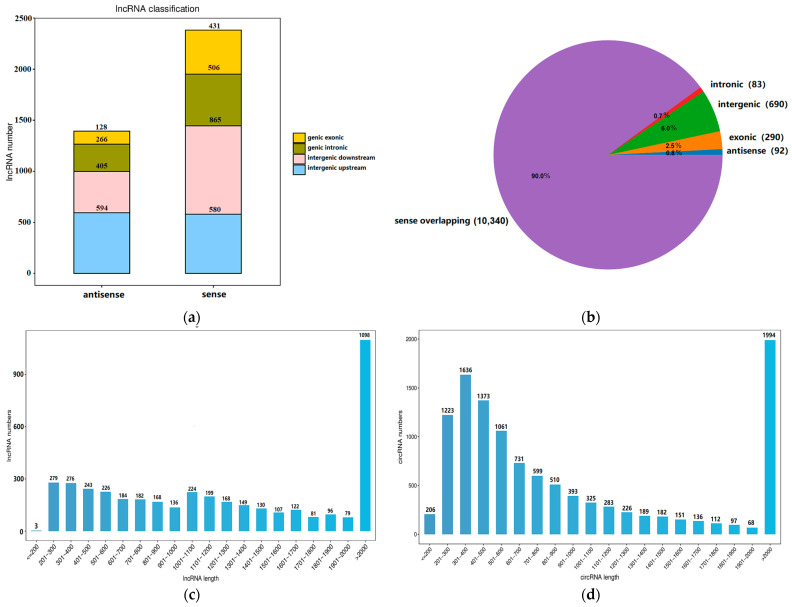

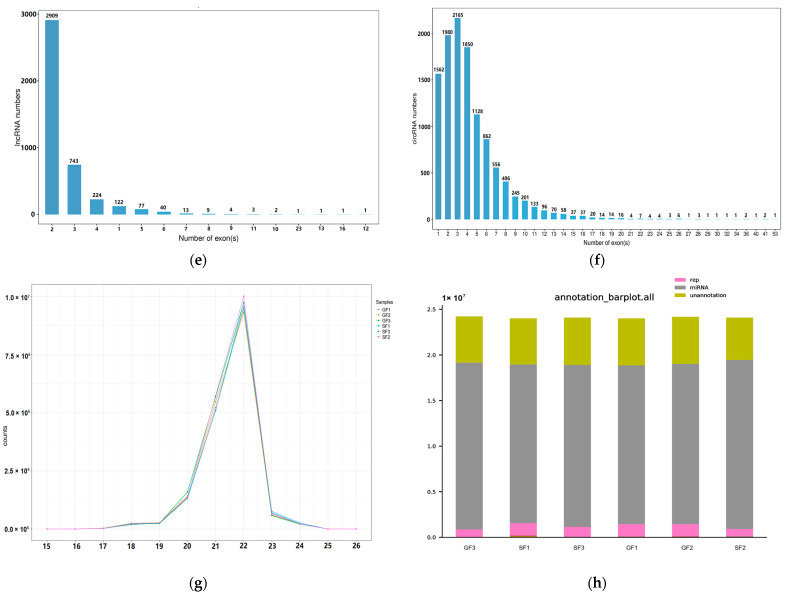
(**a**) Statistics of lncRNA types. (**b**) Statistics of circRNA types. (**c**) Distribution map of lncRNA sequence lengths. The horizontal and vertical axes represent lncRNA length and number, respectively. (**d**) Distribution map of circRNA sequence lengths. The horizontal and vertical axes represent circRNA length and number, respectively. (**e**) Statistics of lncRNA exon numbers. The horizontal and vertical axes represent the number of exons in lncRNAs and the number of lncRNAs, respectively. (**f**) Distribution map of circRNA exon numbers. The horizontal and vertical axes represent the number of exons contained in circRNAs and the number of circRNAs, respectively. (**g**) Line diagram showing the length distribution of known miRNAs. (**h**) Bar chart showing the category notes of small RNAs. The horizontal and vertical axes represent each fat sample and the read counts for each small RNA type, respectively.

A total of 17,269 mRNAs, 4150 lncRNAs, 11,495 circRNAs, and 1468 miRNAs were identified in yak fat. The total length of the identified lncRNAs was 6,719,170 nt, and the average length was 1619.08 nt. The total length of the circRNAs was 38,752,008 nt, and the average length was 3371.21 nt. The identified miRNAs contained 726 known miRNAs and 742 newly predicted miRNAs. The numbers of exonic antisense, intronic antisense, intergenic downstream antisense, intergenic upstream antisense, exonic sense, intronic sense, intergenic downstream sense, and intergenic upstream sense for the lncRNA transcripts were 128, 266, 405, 594, 431, 506, 865, and 580, respectively (Figure 1a). Sense-overlapping circRNAs accounted for 90% of the circRNAs, whereas intergenic circRNAs accounted for 10% (Figure 1b). The length of most lncRNAs containing two exons was more than 2000 bp (Figure 1c). The length of most circRNAs containing less than six exons was 201–600 bp or longer than 2000 bp (Figure 1d). Most lncRNAs contained two exons (Figure 1e), and most circRNAs contained one to five exons (Figure 1f). The length of the most well-known miRNAs was 22 (Figure 1g). A column chart of classification notes for small RNAs is shown in Figure 1h.

### 2.3. Differential Expression RNAs in Yak Subcutaneous Fat

**Figure 2:**
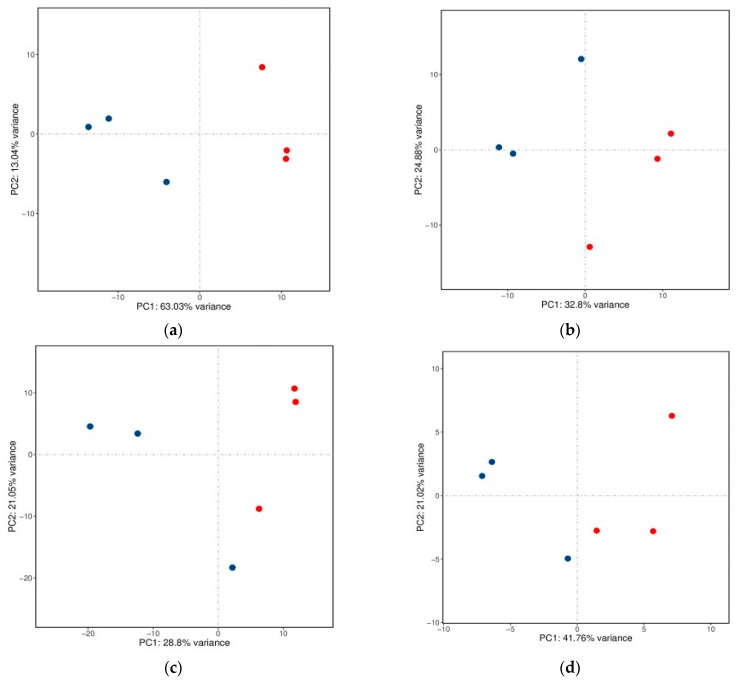
(**a**) Principal component analysis (PCA) score plot for mRNA expression in yak subcutaneous fat. Red dots represent the fat samples from the stall feeding (SF) group; blue dots represent the fat samples from the grazing (GF) group. (**b**) PCA score plot for lncRNA expression in yak subcutaneous fat. (**c**) PCA score plot for circRNA expression in yak subcutaneous fat. (**d**) PCA score plot of miRNA expression in yak subcutaneous fat.

**Figure 3:**
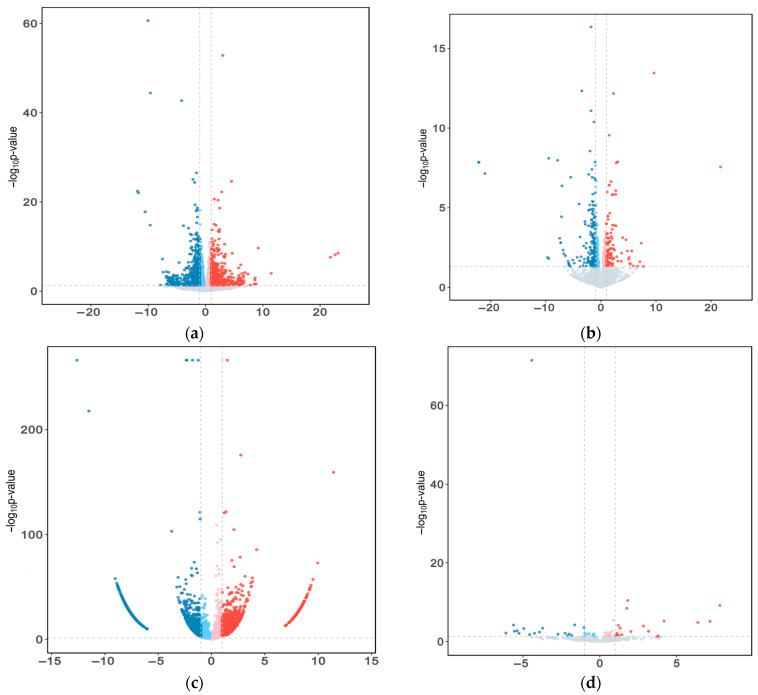
(**a**) Volcano plot of differentially expressed (DE) mRNAs in yak subcutaneous fat (SF vs. GF). The X-axis represents the log_2_ fold change (FC) values. Blue and red dots represent the down- and up-regulated DE mRNAs in the SF group, respectively. Grey dots represent no significant difference. (**b**) Volcano plot of DE lncRNAs in yak subcutaneous fat (SF vs. GF). Blue and red dots represent the down- and up-regulated DE lncRNAs in the SF group, respectively. (**c**) Volcano plot of DE circRNAs in yak subcutaneous fat (SF vs. GF). Blue and red dots represent down- and up-regulated DE lncRNAs in the SF group, respectively. (**d**) Volcano plot of DE miRNAs in yak subcutaneous fat (SF vs. GF). Blue and red dots represent the down- and up-regulated DE miRNA in the SF group, respectively.

The principal component analysis (PCA) score plots for the mRNA, lncRNA, circRNA, and miRNA expression in yak fat under the two feeding systems are shown in Figure 2a–d, respectively. The yak fat samples were divided into two different groups, an SF group and a GF group, showing significant differences in RNA expression between them. Volcano plots of the DE mRNAs, DE lncRNAs, DE circRNAs, and DE miRNAs are shown in Figure 3a–d, respectively. The expression of 383 mRNAs was down-regulated in the SF group, whereas the expression of 294 mRNAs was up-regulated. Information about the mRNAs involved in regulating fat deposition is shown in Table 1. The expression of 120 lncRNAs was significantly different between the two groups; of these, 76 lncRNAs were down-regulated in the SF group and 44 were up-regulated. The DE lncRNAs related to fat metabolism mainly included *TCONS00045143*, *TCONS00051664*, *TCONS00011092*, *TCONS00023345*, *TCONS00035557*, *TCONS00039145*, *TCONS00051664,* and *TCONS00027358*. The expression of 2216 circRNAs differed significantly between the SF and GF groups. Among them, 1357 circRNAs were down-regulated in the SF group, whereas 859 circRNAs were up-regulated. The circRNAs involved in lipid metabolism mainly included *circRNA05148*, *circRNA10119*, *circRNA10117*, *circRNA06092*, *circRNA10545*, *circRNA06097*, *circRNA04135*, *circRNA07932*, *circRNA05655*, *circRNA06099*, *circRNA01572*, *circRNA02163*, *circRNA10118*, *circRNA04137*, *circRNA09996*, *circRNA03334*, *circRNA06093*, *circRNA00666*, *circRNA04167*, *circRNA04629*, *circRNA06275*, *circRNA04415*, and *circRNA01040*. The expression of 15 miRNAs differed significantly between the SF and GF groups. Among them, six miRNAs were down-regulated in the SF group, whereas nine miRNAs were up-regulated.

### 2.4. Gene Ontology (GO) Analysis for Differentially Expressed (DE) mRNAs in Yak Subcutaneous Fat

**Figure 4:**
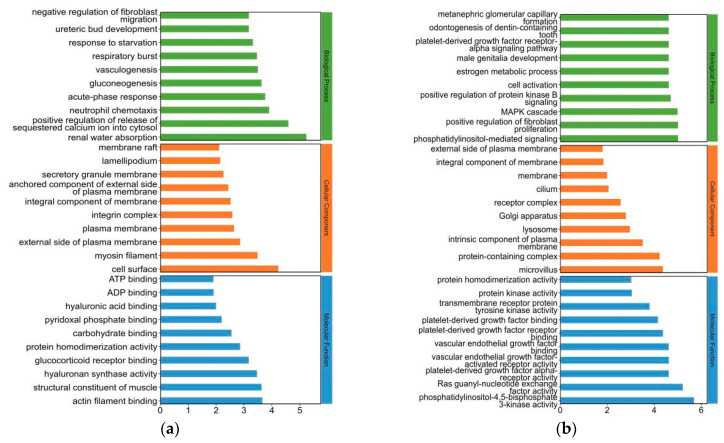

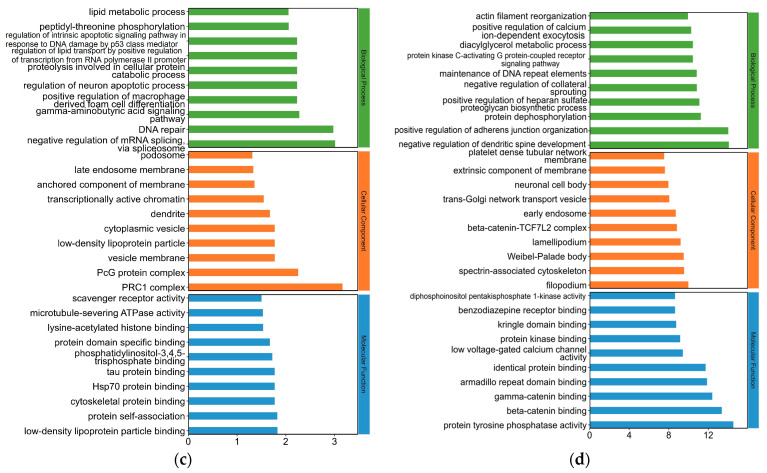
(**a**) Histogram of Gene Ontology (GO) terms for DE mRNA enrichment. The horizontal and vertical axes represent the term name and −log_10_ *p*-value, respectively. Green, red, and blue terms refer to the biological process, cellular component, and molecular function, respectively. (**b**) Histogram of GO terms for the enrichment of the contiguous genes of DE lncRNAs. (**c**) Histogram of GO terms for the enrichment of the host genes of DE circRNAs. (**d**) Histogram of GO terms for the enrichment of the target genes of DE miRNAs.

The GO term enrichment information for DE mRNAs, DE lncRNAs, DE circRNAs, and DE miRNAs is shown in Tables S2–S5, respectively. A histogram of the top 30 GO terms for DE RNAs is shown in Figure 4. GO terms associated with DE mRNA enrichment that were related to fat deposition mainly included positive regulation of the release of sequestered calcium ion into cytosol and gluconeogenesis. GO terms related to the enrichment of host genes of DE lncRNAs in fat deposition mainly included phosphatidylinositol-mediated signaling, positive regulation of fibroblast proliferation, MAPK cascade, positive regulation of protein kinase B signaling, and regulation of mesenchymal stem cell differentiation. GO terms associated with enrichment of host genes of DE circRNAs in fat deposition mainly included the negative regulation of mRNA splicing, via the spliceosome, proteolysis involved in cellular protein catabolic processes, regulation of lipid transport by the positive regulation of transcription from RNA polymerase II promoter, the lipid metabolic process, cholesterol transport, positive regulation of the low-density lipoprotein particle receptor biosynthetic process, lipoprotein transport, and the positive regulation of cholesterol storage. GO terms related to the enrichment of target genes of DE miRNAs in fat deposition mainly included the diacylglycerol metabolic process, positive regulation of adherens junction organization, positive regulation of calcium ion-dependent exocytosis, lipid phosphorylation, and regulation of the hormone metabolic process.

### 2.5. Kyoto Encyclopedia of Genes and Genomes (KEGG) Analysis for DE RNAs in Yak Subcutaneous Fat

**Figure 5:**
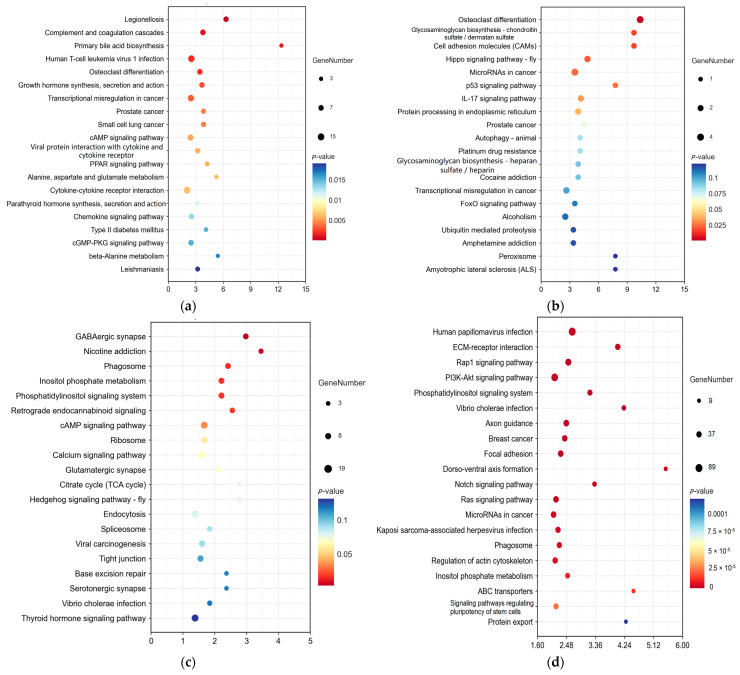
(**a**) Bubble diagram of the top 20 Kyoto Encyclopedia of Genes and Genomes (KEGG) terms for DE mRNAs. The horizontal axis represents the enrichment score. The larger the item bubble, the more DE mRNAs the item contains. As the bubble’s color changes (purple–blue–green–red), the enrichment value gradually dwindles, and the difference becomes more significant. (**b**) Bubble diagram of the top 20 KEGG terms for the host genes of DE lncRNAs. (**c**) Bubble diagram of the top 20 KEGG terms for the host genes of DE circRNAs. (**d**) Bubble diagram of the top 20 KEGG terms for the target genes of DE miRNAs.

The KEGG enrichment terms for DE mRNAs, DE lncRNAs, DE circRNAs, and DE miRNAs are shown in Tables S6–S9, respectively. The bubble diagrams of the top 20 KEGG terms for DE RNAs are shown in Figure 5. The KEGG enrichment terms for the DE mRNAs that were related to fat deposition mainly included primary bile acid biosynthesis, bile secretion and action, the cAMP signaling pathway, the PPAR signaling pathway, alanine, aspartate, and glutamate metabolism, cytokine–cytokine receptor interaction, and the cGMP–PKG signaling pathway. The KEGG enrichment terms for the host genes of DE lncRNAs related to fat deposition mainly included protein processing in the endoplasmic reticulum. The KEGG enrichment terms for the host genes of DE circRNAs associated with fat deposition mainly included the phagosome, inositol phosphate metabolism, phosphatidylinositol signaling system, cAMP signaling pathway, and ribosome and calcium signaling pathway. The KEGG enrichment terms for the target genes of DE miRNAs related to fat deposition mainly included ECM–receptor interaction, the rap1 signaling pathway, the PI3K–Akt signaling pathway, the phosphatidylinositol signaling system, axon guidance, focal adhesion, the phagosome, regulation of the actin cytoskeleton, and inositol phosphate metabolism. The KEGG enrichment statistics for crucial signaling pathways are shown in Table 2.

### 2.6. Co-Expression RNA (CeRNA) Regulatory Network for DE RNAs in Yak Subcutaneous Fat

A total of 931 miRNA–mRNA relationship pairs, 31 miRNA–lncRNA relationship pairs, and 215 miRNA–circRNA relationship pairs were obtained in this study. Among the ceRNA networks—mRNAs–miRNAs–circRNAs and mRNAs–miRNAs–lncRNAs—four miRNAs (*bta-miR-339b*, *bta-miR-205*, *bta-miR-2316*, and *bta-miR-2892*) targeted more than 100 genes. The eight DE mRNAs involved in fat metabolism were *UNC119*, *ACSS2*, *TNFRSF21*, *MCAT*, *NR4A3*, *MAST3*, *SHOX*, and *ACSF3*. Target prediction analysis for DE miRNAs and DE lncRNAs showed targeting relationships between *bta-miR-2316* and *TCONS00042948* and *TCONS00012083*. Targeting relationships were also identified between *bta-miR-2892* and *circRNA03372*, *circRNA06073*, and *circRNA06078*. For DE miRNAs and DE circRNAs, target prediction showed interactions between *bta-miR-2316* and *circRNA00274*, *circRNA00679*, *circRNA01768*, *circRNA05277*, *circRNA06073*, *circRNA06078*, *circRNA06528*, *circRNA07560*, *circRNA07750*, *circRNA08402*, *circRNA10133*, *circRNA_10476*, and *circRNA11309*. Information about the important DE lncRNAs, circRNAs, and miRNAs regulating yak fat deposition is shown in Table 3, Table 4 and Table 5, respectively. The method for screening important mRNAs, lncRNAs, miRNAs, and cirRNAs was as follows: GO and KEGG analyses were performed for DE mRNAs, DE lncRNAs, DE miRNAs, and DE cirRNAs. Then, GO and KEGG terms related to fat metabolism or adipocyte proliferation and differentiation were identified. Finally, mRNAs, lncRNAs, miRNAs, and cirRNAs present in both the screened GO terms and KEGG pathways and the CeRNA regulatory network were chosen as the important genes and ncRNAs.

### 2.7. Comparative Results of Transcriptome and Quantitative Reverse-Transcription PCR (qPCR) for RNAs

**Figure 6:**
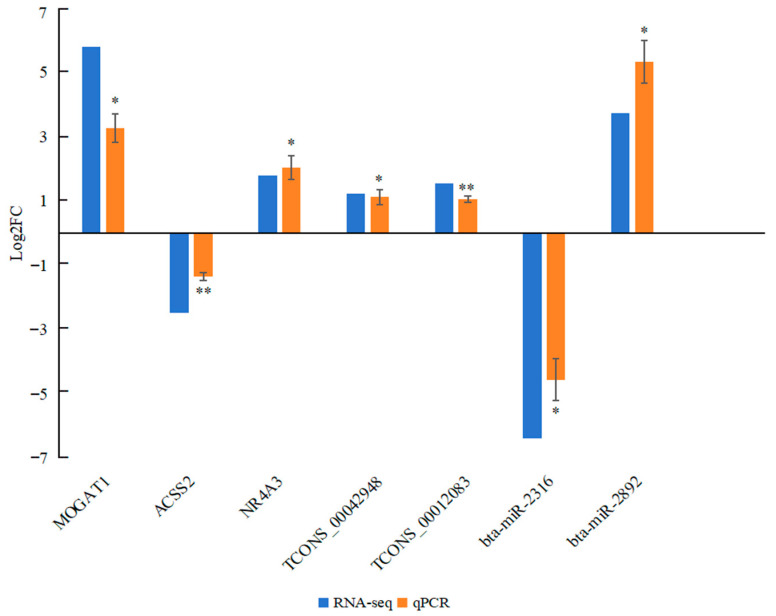
Comparison of DE mRNA and ncRNA expression levels between the SF and GF groups based on whole-transcriptome and *qPCR* analyses. * *p* < 0.05 and ** *p* < 0.01.

The RNAs *bta-miR-2316*, *bta-miR-2892*, *MOGAT1*, *ACSS2*, *NR4A3*, *TCONS_00042948*, and *TCONS_00012083* were selected for qPCR analysis. The comparative results of RNA expression levels between transcriptome and qPCR analyses are shown in Figure 6. The qPCR analysis revealed significant differences in the expression of the selected RNAs between the GF and SF groups. The expression levels of *NR4A3*, *MOGAT1*, *TCONS_00042948*, *TCONS_00012083*, and *bta-miR-2892* were up-regulated in the SF group, whereas *ACSS2* and *bta-miR-2316* were down-regulated. All seven DE mRNAs and ncRNAs exhibited similar expression patterns in comparison to the RNA-seq data, indicating the reliability of the RNA-seq results for whole-transcriptome analysis.

## 3. Discussion

Our previous study [6] showed that the subcutaneous fat thickness in the back and waist of SF yaks was significantly greater than in GF yaks, indicating that the SF system can significantly improve fat deposition in yaks. The TMR diets possessed a higher nutritional value, with greater fat and protein contents than grass. Moreover, the carbohydrates in TMR were more easily absorbed by yaks. Fatty acids, amino acids, and carbohydrates can undergo mutual transformation through the TCA cycle [18,19]. Compared to grass, the higher fat, carbohydrate, and protein contents in TMR can be easily converted into short-chain fatty acids, which are subsequently transformed into long-chain SFAs and MUFAs. KEGG enrichment analysis of DE mRNAs showed that the differences in fat deposition between the two feeding systems were mainly related to growth hormone synthesis, secretion, and action, the PPAR signaling pathway, cytokine–cytokine receptor interaction, and the cGMP–PKG signaling pathway. These pathways are mainly involved in fat and glucose metabolism. The cGMP–PKG signaling pathway regulates glucose tolerance [20], and glucose and fat metabolism are interconnected through the tricarboxylic acid cycle. The PPAR signaling pathway is very critical for fat deposition, and no single factor can independently start lipogenesis in the absence of PPARγ. The DE mRNAs directly enriched in the PPAR signaling pathway included *EHHADH*, *GK2*, *THNSL2*, and *ACOX2*. The functions of these four genes are mainly related to fatty acid transport, fatty acid oxidation, and gluconeogenesis. It has been verified that *EHHADH* and *ACOX2* can regulate cattle fat metabolism [21,22]. Moreover, GO analysis showed that *MOGAT1*, *ELOVL6*, *GPD1*, *APOC3*, *FASN*, *LIPK*, *LIPA*, *SCD*, *UNC119*, *ACSS2*, *TNFRSF21*, *MCAT*, *NR4A3*, *MAST3*, *SHOX*, and *ACSF3* are all related to fat metabolism in yaks. Some of these genes are candidate genes for fat synthesis [23,24], triglyceride transport [25], and fatty acid synthesis [26,27,28] in the adipose tissue of cattle or cows. Therefore, the PPAR and cGMP–PKG signaling pathways are very important in regulating fat deposition in yaks through specific genes.

Bovine lncRNAs exhibit limited capacity for protein coding, obvious tissue specificity, and low conservation characteristics [29]. Some crucial lncRNAs involved in regulating fat metabolism in cattle and cows have been found [30,31]. However, studies on lncRNAs related to cattle fat are relatively fewer compared to model organisms, and research on lncRNAs in yak fat is incredibly rare. The functions of DE lncRNAs in yak subcutaneous fat under SF and GF are relatively complex and diverse, with only a few DE lncRNAs involved in the regulation of lipid metabolism and transcription. Moreover, DE lncRNAs are enriched in the Hippo signaling pathway, which plays an important role in adipocyte proliferation [32]. Therefore, lncRNAs may influence yak fat deposition, primarily by affecting adipocyte proliferation. The current studies on circRNA regulation of fat metabolism mainly focus on miRNA adsorption by circRNA sponges. CircRNAs affect fatty acid oxidation, transport, synthesis, gluconeogenesis, and glycolysis, regulating fat deposition in cattle and cows through the PPAR signaling pathway [33,34]. Additionally, circRNAs promote the expression of genes associated with preadipocyte differentiation in bovine animals [35] by combining miRNAs. GO enrichment terms for DE circRNAs were mainly related to cholesterol and lipid transport and metabolism. Moreover, DE circRNAs are significantly enriched in the cAMP signaling pathway, which is closely related to cell fate and lipid metabolism in cattle [33,36]. GO analysis showed that many DE circRNAs in yak subcutaneous fat are involved in regulating fat metabolism, but most DE circRNAs in yaks differ from those found in cattle. The regulation of circRNAs in yak fat deposition under different feeding systems is also complicated. Researchers have identified *miR-378*, *miR-27b*, *miR-320a*, *miR-2400*, and *miR-23a* as regulators of fat metabolism [37]. KEGG enrichment analysis of target genes for DE miRNAs highlighted pathways related to adipocyte proliferation, glycometabolism, and transcription regulation. The PI3K–Akt signaling pathway is involved in fat deposition and metabolism in cattle [38], cows [39], and yaks [40]. These results align with the KEGG enrichment results for DE lncRNAs and DE microRNAs. Therefore, the main functions of DE RNAs in yak fat under SF and GF feeding systems focus on regulating fat metabolism and adipocyte proliferation.

The different feeding systems can provide yaks with different energy levels and nutrient substances, which have a significant impact on gene expression. Different nutrients and energy levels are closely related to the regulation of metabolic signaling pathways and can activate or inhibit the transcription level of specific genes. The PI3K–Akt, PPAR, and cAMP signaling pathways are all involved in the regulation of many basic cellular processes and can be affected by different energy levels and nutrient substances. SF yaks can obtain more nutrient substances from TMR than GF yaks, leading to differences in the PI3K–Akt, PPAR, and cAMP signaling pathways in yak fat under the two feeding systems. These differences in RNA expression and the regulatory networks between mRNAs and ncRNAs are further modulated by the three signaling pathways.

**Figure 7:**
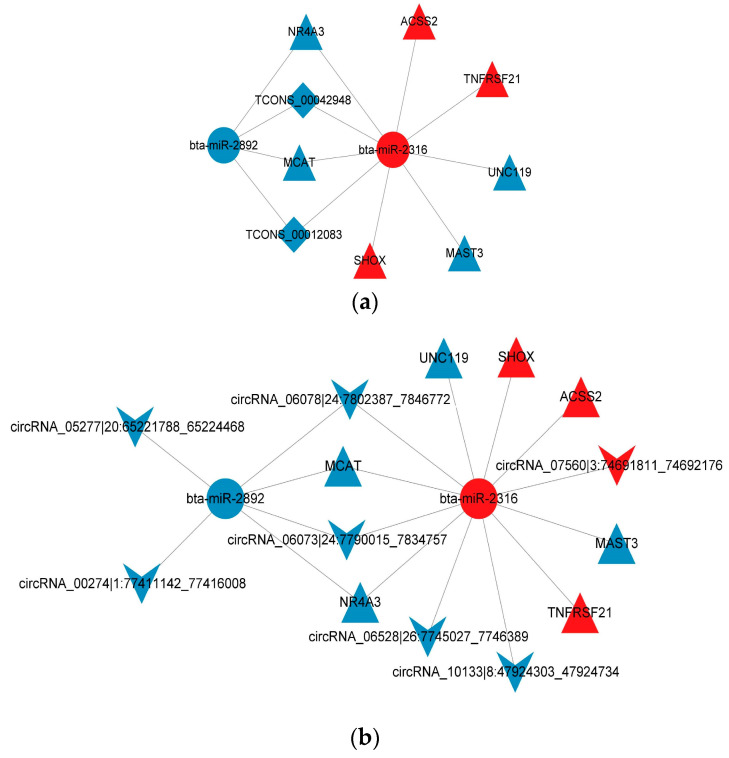
(**a**) Network diagram of the co-expression RNAs (ceRNAs) for the crucial mRNAs, miRNAs, and lncRNAs in yak fat. Circles, triangles, arrows, and rhombuses represent the crucial miRNAs, mRNAs, circRNAs, and lncRNAs, respectively; the lines represent the regulatory relationships. (**b**) Network diagram of the ceRNAs for the crucial mRNAs, miRNAs, and circRNAs.

Many mRNAs, lncRNAs, and circRNAs can regulate lipogenesis through interactions with co-expression networks. The results of the ceRNA regulatory network analysis showed that *TCONS00042948*, *TCONS00012083*, *circRNA00274*, *circRNA05277*, *circRNA06073*, *circRNA06078*, *circRNA06528*, *circRNA07560*, and *circRNA10133* targeted *bta-miR-2316*, while *bta-miR-2316* targeted the *MCAT*, *ACSS2*, *SHOX*, and *NR4A3* genes. Additionally, *circRNA06073* and *circRNA06078* targeted *bta-miR-2892*, which targeted the *ACSF3* gene (Figure 7a,b). It has been reported that the MCAT gene can regulate lipid metabolism in cows [41] and cattle [42]. The *ACSS2* gene plays an important role in carbon metabolism, fatty acid production, and oxidation. Its expression is regulated by transcription factors that activate the genes involved in cholesterol and unsaturated fatty acid synthesis [43,44]. *ACSS2* has also been reported to positively regulate fatty acid synthesis in cattle [45] and cows [46]. The *SHOX* gene regulates cattle intramuscular fat deposition [47], and the *ACSF3* gene is a useful molecular marker for cattle fat deposition [48]. The *NR4A3* gene affects insulin sensitivity, further regulating fat deposition [49]. Moreover, there may be an upstream or downstream regulatory relationship among the *MCAT*, *ACSS2*, *SHOX*, *ACSF3*, and *NR4A3* genes, and the *EHHADH*, *GK2*, *THNSL2*, and *ACOX2* genes were found to be enriched in the PPAR signaling pathway.

The target pathways of *bta-miR-2316* are related to adipogenesis [50,51]. Moreover, it has been verified that *bta-miR-2316* regulates bovine lipid metabolism and fat deposition [52,53]. *bta-miR-2892* is also closely related to subcutaneous fat deposition in cattle [52]. However, the regulatory mechanisms of *bta-miR-2316* and *bta-miR-2892* in bovine fat deposition have not yet been reported. Based on these findings, it was inferred that *bta-miR-2316* and *bta-miR-2892* are crucial genes regulating yak fat deposition. Several circRNAs—including *TCONS00042948*, *TCONS00012083*, *circRNA00274*, *circRNA05277*, *circRNA06073*, *circRNA06078*, *circRNA06528*, *circRNA07560*, and *circRNA10133*—were found to affect the expression of *MCAT*, *ACSS2*, *SHOX*, and *NR4A3* by interacting with *bta-miR-2316* in yak fat. In addition, *circRNA06073* and *circRNA06078* also affected the expression of the *ACFS3* gene through interaction with *bta-miR-2892* in yak fat. Meanwhile, an inverse expression trend was observed between *bta-miR-2316* and both *MCAT* and *NR4A3*. Because studies on the effect of ncRNAs in fat metabolism are still rare, the specific regulatory mechanisms of many miRNAs, lncRNAs, and circRNAs in the fat deposition of livestock remain unclear, with only limited references currently available. In the future, these screened ncRNAs in yak adipose tissue will be further studied, and particular attention will be paid to the control axis *TCONS00042948*/*TCONS00012083*/*bta-miR-2316/MCAT*/*NR4A3* in yak fat deposition. At present, research on the regulatory relationship between mRNAs and ncRNAs in yaks is almost absent, and the goal of molecular breeding efforts is to mainly focus on mRNAs. This study found crucial ncRNAs, proposed a potential regulatory pathway for fat deposition, and provided new insights for yak genetic breeding.

## 4. Materials and Methods

### 4.1. Animals and Sample Collection

The animal experiment was carried out in Qinghai Province, China. Six healthy male yaks (two years old, 210.33 ± 10.23 kg, Huanhu yak) from the same population were used as experimental animals. All yaks were randomly divided into the GF and SF groups, with each group containing three yaks. The GF yaks were grazed in natural pasture from 07:00 to 19:00 every day and were allowed to freely eat grass and drink water; the SF yaks were fed with total mixed ration (TMR) twice a day and could freely drink water. Grass samples were collected from ten different areas in the pasture. After being weighed, the grass samples were dried at 65 °C in an air-dry oven and then milled through a 5 mm screen and stored. The ether extract, calcium, phosphorus, neutral detergent fiber, and acid detergent fiber contents in the grass (dry matter) and TMR were determined according to the Association of Official Analytical Chemists (AOAC) International Official Methods. The crude protein content in the grass was determined using the semi-micro Kjeldahl method. The fatty acid profiles were determined according to the Chinese standard GB/T17377-2008 [54]. A total of 18 fatty acids were detected, and the main fatty acids both in the grass and TMR were C16:0, C18:0, C18:1, C18:2n6, and C18:3n3. The composition, common nutrition, and main fatty acid content in the grass and TMR are shown in Table S10. The feeding test lasted six months, after which all of the yaks were humanely harvested at a commercial abattoir in the morning after being fasted for 24 h and cut off from water for 8 h. Subcutaneous fat samples (12th–13th rib level) were collected and stored in liquid nitrogen at once.

### 4.2. Total RNA Extraction

Total RNA in yak fat was extracted using a mirVana miRNA Isolation Kit (Ambion, Austin, TX, USA). Fat samples were homogenized with 600 μL of binding buffer, after which 30 μL of miRNA homogenate additive was added. The mixture was fully mixed and left to stand in an ice bath for 10 min. Phenol and chloroform were added, and the mixture was centrifuged for 5 min. The supernatant was transferred out, and 1.25 times the volume of 100% ethanol was added. The mixture was added to a column and then centrifuged at 13,000 r/min for 30 s. The supernatant was discarded, and 350 μL of miRNA wash solution 1 was added to the centrifuge column. The mixture was centrifuged at 13,000 r/min for 30 s, then the supernatant was discarded, and the centrifuge column was weighed. The centrifuge column was placed in the collection tube once again. Next, 10 μL of DNase I and 70 μL of Buffer RDD QIAGE N were added to the membrane in the column, and the column was left to stand at 25 °C for 15 min. Following this, 350 μL of miRNA wash solution 1 was added to the centrifuge column once again, and the mixture was centrifuged at 13,000 r/min for 30 s. The supernatant was discarded, and then the centrifuge column was weighed and placed in the collection tube once again. Next, 500 μL of wash solution 2/3 was passed through the column twice, and the column was centrifuged at 13,000 r/min for 30 s. The supernatant was discarded, and the centrifuge column was placed in the collection tube once again. The column was centrifuged for 1 min and then put into a new collection tube, after which 100 μL of elution solution preheated at 95 °C was added into the column center before the column was left to stand for 2 min. The mixture was centrifuged at 25 °C for 20 s, and the resulting liquid in the tube was the total RNA. The RNA integrity and purity were analyzed using a Tanon 2500 agarose gel electrophoresis imager and an ultraviolet spectrophotometer (Nanodrop 2000, Thermo, Waltham, MA, USA), respectively.

### 4.3. Sequencing and Raw Data Analysis

#### 4.3.1. Sequential Detection

Transcriptome sequencing was performed by OE Biotech Co. (Shanghai, China). The deribosome RNA sequencing library was constructed using a TruSeq Stranded Total RNA Library Prep Kit with Ribo-Zero Gold (Illumina, Ipswich, MA, USA; Cat. No. RS-122-2301). Library quality was measured using the Agilent 2100 bioanalyzer. Finally, the library was sequenced on the HiseqTM 2500 instrument (Illumina Corp., San Diego, CA, USA), and 150 nt paired-end reads were obtained. Meanwhile, 1 µg of total RNA was transferred out, and a TruSeq Small RNA Sample Prep Kit (Illumina, San Diego, CA, USA; Cat. No. RS-200-0012) was used to construct the small RNA library. The library quality was monitored using Agilent Bioanalyzer 2100 (Santa Clara, CA, USA), and the small RNA libraries were detected using Illumina HiSeq^TM^ 2500 (San Diego, CA, USA).

#### 4.3.2. Raw Data Quality Control, Comparison, and Stitching

SortMeRNA (version 4.2.0) and Trimmomatic software (version 0.39) were used to remove residual rRNA sequences, adaptors, and low-quality reads from the sequences. Next, the quality of the obtained reads was detected using fastp (version v0.11.5, parameters: length_required 50) software. The clean reads were compared to the yak reference genome using HISAT2 (version 2.0.5, parameters:--rna-strandness-rf--fr). The reads compared to the yak genome were assembled and spliced using StringTie (version 1.3.3b, parameters:--rf), and then the transcriptomes of all samples were merged, and the comprehensive transcriptomes were reconstructed using Perl scripts.

#### 4.3.3. Identification of lncRNAs and circRNAs

The results of the transcript assembly were screened. First, merged transcripts were compared to reference transcripts using cuffcompare (version 2.2.1), and the location type of the remaining transcripts was confirmed. The transcripts annotated as “i”, “u”, “x”, and “o” were retained. Next, transcripts longer than 200 bp and containing more than 2 exons were retained. Finally, the coding potential of the remaining transcripts was predicted using the Coding Potential Calculator (CPC, score < 0), Coding-Non-Coding-Index (CNCI, score < 0), k-mer scheme (PLEK, score < 0), and Pfam (E-value < 0.001), and these transcripts without coding potential were candidate lncRNAs. CircRNAs were predicted based on the comparison results from BWA using CIRI software (https://sourceforge.net/projects/ciri/) (accessed on 7 March 2024). First, clean reads were compared to the yak reference genome using BWA MEM, and then the SAM file was obtained. Next, balanced junction reads were detected based on XS/HYM or xMysS/H PCC signals. Third, junction reads were filtered by PEM and GT-AG signals. Finally, unbalanced junction reads were detected using the DM algorithm. The partner genes of DE lncRNAs are usually inferred from the positions of lncRNAs on the genome. The lncRNA adjoins or overlaps with protein-coding genes on the chromosome, and then the gene is considered to be the partner gene of the lncRNA. The identification of the host gene of a circRNA was based on the circRNA’s localization on the genome. When the circRNA’s formation area is adjacent to or overlaps with a protein-coding gene on the yak genome, the gene is considered to be the circRNA’s host gene. 

#### 4.3.4. Identification and Prediction of miRNAs

The basic reads were converted into raw reads by base calling. The joint sequence was removed using cutadapt (version 1.14) software, and sequences shorter than 15 nt or longer than 41 nt were removed. Sequences with Q20 above 80% were retained using fastx_toolkit (version 0.0.13) software. Reads containing N bases were filtered using NGSQCToolkit (version 2.3.3). The fastx_toolkit (version 0.0.13) was used to count the number of clean reads, and unique reads were obtained. Clean reads were compared to the yak’s genome, and the percentage of reads matching the yak genome was counted. Clean reads were compared with the Rfam (version 10.0) database using blastn software (version 1.1), and results with an E-value less than 0.01 were extracted, after which rRNA, snRNA, snoRNA, tRNA, and other sequences were annotated. Sequences annotated to the Rfam (version 10.1) (https://ftp.ebi.ac.uk/pub/databases/Rfam/) (accessed on 10 March 2024) database were filtered. Reads matching transcripts less than 15 bp or more than 41 bp were also removed. The filtered sequence was compared to the repeat database using RepeatMasker software (version 2.0.4) to identify repeat sequences, and the identified repeat sequences were filtered. Bowtie software (version 2) was used to perform one-base mismatching filtered reads with the mature miRNA sequences in the miRBase v.21 database (http://www.mirbase.org/) (accessed on 10 March 2024), and the corresponding sequences were considered to be known miRNAs. The unannotated small RNA sequence was used to predict new miRNAs using Mirdeep2 software (version 2), and the secondary structure of new miRNAs was predicted using RNAfold software (version 1.99.3.4).

#### 4.3.5. Differentially Expressed RNA Analysis and Target Genes of DE miRNA Prediction

Htseq-count software (version 0.11.0) was used to calculate the number of reads obtained matching protein-coding genes. Using reference transcripts as a library, the expression abundance of lncRNAs in yak fat was determined via sequence similarity comparison using bowtie software (version 2) and eXpress software (version 4). The fragments per kilobase million (FPKM) values for the expression level of mRNAs and lncRNAs were calculated; circRNAs were quantified by the junction reads per billion mapped reads (RPB); and miRNA expression was calculated by transcript per million (TPM). DESeq2 software (version R3.6) was used to analyze DE mRNAs, DE lncRNAs, DE circRNAs, and DE miRNAs. Fold change (FC) values were calculated, and NB was used to test the significance of the differences. Finally, the DE mRNAs, DE lncRNAs, DE circRNAs, and DE miRNAs were screened according to the FC and *q*-values (SF vs. GF). FC > 2 or <0.5 and *q* < 0.05 were set as the DEG and DE ncRNA thresholds. The 100 kb genes upstream and downstream of the lncRNA sequences were selected as the adjacent genes of the lncRNAs. The circRNAs were generally enriched with their host gene as the functional gene. The adjacent genes of the lncRNAs and the host gene of the circRNAs were obtained by comparing lncRNAs and circRNAs to the yak genome using hisat2 software (version 2.1.0), respectively. The target genes of DE miRNAs were predicted using miranda software (version 0.9.37).

#### 4.3.6. Functional Enrichment Analysis

The potential functions of DE mRNAs, partner genes of DE lncRNAs, host genes of DE circRNAs, and target genes of DE miRNAs were explored using GO and KEGG analyses. The GO annotation information was obtained by comparing the DEG transcript sequences to the Swiss-Prot database (http://www.gpmaw.com/html/swiss-prot.html) (accessed on 12 March 2024). Furthermore, KEGG annotation was obtained by comparing gene transcript sequences to the KASS database (http://www.genome.jp/tools/kaas/) (accessed on 12 March 2024). GO and KEGG terms with *p* < 0.05 were considered significant enrichment terms.

#### 4.3.7. Co-Expression RNA (CeRNA) Networks’ Construction

The co-expression networks were analyzed using Pearson’s correlation via the ropls package in R (version 3.6.2). A correlation coefficient ≥ 0.80 and *p* < 0.05 were set as thresholds. First, the miRNA–lncRNA and miRNA–circRNA co-expression relationships were calculated. The regulatory relationships were predicted based on the screened miRNA–lncRNA and miRNA–circRNA co-expression. Then, the ceRNA MuTATE [55] method was used to calculate the scores among the ceRNA relationship pairs, and the probability of ceRNA relationship pairs sharing some miRNAs was calculated by the hypergeometric distribution algorithm. Finally, the ceRNA relationship pairs with high reliability were obtained. The network diagrams of the ceRNA ternary relationships for mRNA–miRNA–circRNA and mRNA–miRNA–lncRNA were drawn based on the 200 mRNA–miRNA–circRNA relationship pairs among the top 100 mRNA–circRNAs and 200 mRNA–miRNA–lncRNA relationship pairs among the top 100 mRNA–lncRNAs in the ceRNA analysis results, respectively.

### 4.4. qPCR RNA Expression Results

The steps of qPCR for miRNAs were as follows: Each reverse transcription (RT) consisted of 0.5 μg of RNA, 5 μL of 2 × TS miRNA reaction mix, and 0.5 μL of TransScrip miRNA RT Enzyme Mix, with a total volume of 10 μL. Reaction was performed in a GeneAmp^®^ PCR System 9700 (Applied Biosystems, Waltham, MA, USA) for 60 min at 37 °C, followed by the heat inactivation of RT for 5 s at 85 °C. The 10 μL RT reaction mix was diluted to 100 μL in nuclease-free water. Real-time PCR was performed using a LightCycler^®^ 480 Ⅱ Real-time PCR Instrument (Roche, Basel, Switzerland). The 10 μL PCR reaction mixture included 1 μL of cDNA, 5 μL of 2 × PerfectStart™ Green qPCR SuperMix, 0.2 μL of universal primer, 0.2 μL of microRNA-specific primer, and 3.6 μL of nuclease-free water. Reaction was incubated in a 384-well optical plate (Roche, Basel, Switzerland) at 94 °C for 30 s, followed by 45 cycles of 94 °C for 5 s and 60 °C for 30 s. Each sample was run in triplicate. Melting curve analysis was performed to validate the specific generation of the expected PCR product. The expression levels of the microRNAs were normalized to 5S rRNA and calculated using the 2^−ΔΔCt^ method. The steps of the qPCR for the mRNAs and lncRNAs were as follows: Each RT reaction consisted of 0.5 μg of RNA, 2 μL of 5 × TransScript All-in-one SuperMix for qPCR, and 0.5 μL of gDNA Remover, with a total volume of 10 μL. Reactions were performed in a GeneAmp^®^ PCR System 9700 (ABI, Carlsbad, CA, USA) for 15 min at 42 °C, followed by 5 s at 85 °C. The 10 μL RT reaction mix was diluted to 100 μL in nuclease-free water. Real-time PCR and melting curve analysis were the same as in the previous statement. Previous research has indicated that changes in *β-actin* gene expression are strongly linearly correlated with changes in total mRNA in the same yak tissue, especially in adipose tissue. Thus, the *β-actin* gene can be used as a reference gene in yak subcutaneous fat. The expression of the *5S* rRNA gene possesses excellent stability and is often used as a reference gene for miRNAs. Therefore, the *5S* rRNA gene was also selected as the reference gene for the miRNAs in this study. The primer sequences were synthesized by Tsingke Biotech Co.,Ltd. (Beijing, China) based on the NCBI database (Table S11).

## 5. Conclusions

The differences in fat deposition in yaks were closely related to lipogenesis, fatty acid synthesis, fat transport, and adipocyte proliferation in adipose tissue. The PPAR, PI3K–Akt, and cAMP signaling pathways were crucial in the regulation of fat deposition in yaks. In the field of molecular breeding, the *MCAT*, *ACSS2*, *ACFS3*, *SHOX*, and *NR4A3* genes are considered candidate genes for fat characteristics in yaks. Our study suggests that the regulatory pathway based on mRNAs and ncRNAs—*TCONS00042948*, *TCONS00012083*/*bta-miR-2316*/*MCAT*, and *NR4A3*—may play an important role in this regulation. This study revealed the overall transcription characteristics of yak fat under different feeding systems, thoroughly identified important regulatory genes and ncRNAs involved in yak fat deposition, and provided new insights for yak genetic breeding from the perspective of the interaction between coding RNAs and ncRNAs.

## Figures and Tables

**Table 1:** Information on the important differentially expressed mRNAs (DE mRNAs) in yak subcutaneous fat under grazing (GF) and stall feeding (SF).

Gene Symbol	Description	log_2_ Fold Change	*q*-Value
*MOGAT1*	Monoacylglycerol O-acyltransferase 1	−5.78	3.76 × 10^−2^
*ELOVL6*	ELOVL fatty acid elongase 6	2.44	8.33 × 10^−12^
*GPD1*	Glycerol-3-phosphate dehydrogenase 1	1.06	5.27 × 10^−3^
*APOC3*	Apolipoprotein C3	21.80	3.11 × 10^−6^
*FASN*	Fatty acid synthase	3.30	2.01 × 10^−3^
*LIPK*	Lipase family member K	−1.98	1.04 × 10^−2^
*LIPA*	Lysosomal acid lipase	−1.54	5.13 × 10^−4^
*SCD*	Stearoyl-CoA desaturase	2.77	1.96 × 10^−4^
*UNC119*	Unc-119 lipid binding chaperone	−1.11	1.26 × 10^−5^
*ACSS2*	Acyl-CoA synthetase short-chain family member 2	2.49	2.68 × 10^−16^
*TNFRSF21*	TNF receptor superfamily member 21	1.09	9.84 × 10^−5^
*MCAT*	Malonyl-CoA-acyl carrier protein transacylase	−0.64	1.03 × 10^−2^
*NR4A3*	Nuclear receptor subfamily 4 group A member 3	−1.79	5.18 × 10^−5^
*MAST3*	Microtubule-associated serine/threonine kinase 3	−1.21	3.14 × 10^−4^
*SHOX*	Short-stature homeobox	6.11	1.73 × 10^−2^

**Table 2:** The KEGG enrichment statistics for the crucial signaling pathways related to the regulation of fat deposition in yaks.

Pathway	List Hits	*p*-Value	Enrichment Score
PPAR signaling pathway	5	5.88 × 10^−3^	4.24
PI3K–Akt signaling pathway	76	1.91 × 10^−10^	2.12
cGMP–PKG signaling pathway	8	1.50 × 10^−2^	2.48

**Table 3:** Information on the important DE lncRNAs in yak subcutaneous fat under GF and SF.

lncRNA ID	Direction	lncRNA Symbol	Target mRNA
*TCONS00042948*	Sense	XLOC012001	ENSBGRT00000031389
*TCONS00012083*	Sense	XLOC003393	ENSBGRT00000038536

**Table 4:** Information on the important DE circRNAs in the yak subcutaneous fat under GF and SF.

circRNA	circRNA Length	Type	Transcript Position	Gene
*circRNA03372*	796	Exonic	16:60894938_60921423	*SH2B3*
*circRNA06073*	39,774	Sense-overlapping	24:7788337_7802802	*STK19*
*circRNA06078*	44,254	Sense-overlapping	24:7788337_7802802	*STK19*
*circRNA00274*	380	Sense-overlapping	1:77318523_77430765	*PAK2*
*circRNA00679*	548	Sense-overlapping	1:172082712_172194902	*KAT2B*
*circRNA01768*	1345	Sense-overlapping	12:1412261_1546561	*USP6NL*
*circRNA05277*	558	Sense-overlapping	20:65208274_65233932	*LIPE*
*circRNA06073*	39,774	Sense-overlapping	24:7788337_7802802	*STK19*
*circRNA06078*	44,386	Sense-overlapping	24:7788337_7802802	*STK19*
*circRNA06528*	699	Sense-overlapping	26:7744912_7746357	*ZNHIT1*
*circRNA07560*	366	Intronic	3:74687137_74695810	*RABGGTB*
*circRNA07750*	2067	Sense-overlapping	3:109743508_109751164	*TOE1*
*circRNA08402*	1018	Sense-overlapping	5:11880919_12172221	*PLEKHA5*
*circRNA10133*	432	Exonic	8:47906904_47933260	*CSNK1G2*
*circRNA10476*	28,908	Sense-overlapping	9:8107091_8173022	*SPOUT1*
*circRNA11309*	53,606	Sense-overlapping	X:93853225_93854235	*UXT*

**Table 5:** Information on the important DE miRNAs in yak subcutaneous fat under GF and SF.

miRNA ID	Sequence	miRNA Length	Target mRNA (Predict)
*bta-miR-2892*	ACTCCGGCCTGGACTGCGGCGGG	23	*ACSF3*
*bta-miR-2316*	GGCGACGGAGGCGCGACCCCCC	22	*UNC119*, *ACSS2*, *TNFRSF21*, *MCAT*, *NR4A3*, *MAST3*, *SHOX*

## Data Availability

The datasets generated for this study can be found in the Sequence Read Archive (https://www.ncbi.nlm.nih.gov/sra) (accessed on 20 May 2025) at NCBI, with the BioProject IDs PRJNA1152715 and PRJNA1264695.

## Supplementary Materials

The following supporting information can be downloaded at: https://www.mdpi.com/article/10.3390/ijms26115359/s1.
