# Supplementary material for: Correlation of Coding and Non-Coding RNAs on the Fat Deposition of Yaks Under Different Feeding Systems
Source: Int J Mol Sci. 2025 Jun 3;26(11):5359. doi: 10.3390/ijms26115359 (PMC12154009; doi:10.3390/ijms26115359)
Supplement: Supplementary file 1 [file ijms-26-05359-s001.zip › Table S1 The preprocessing results of sequencing data quality..pdf]

Table S1. The preprocessing results of sequencing data quality.

Sample	RawReads	RawBases	CleanReads	CleanBases	ValidBases	Q30	GC
GF1	84.70M	12.70G	83.92M	12.35G	97.20%	94.06%	50.86%
GF2	91.80M	13.77G	91.04M	13.44G	97.62%	93.98%	50.71%
GF3	82.51M	12.38G	81.70M	12.01G	97.05%	93.97%	51.11%
SF1	85.60M	12.84G	84.83M	12.51G	97.44%	93.95%	52.30%
SF2	88.25M	13.24G	87.45M	12.90G	97.43%	94.03%	52.14%
SF3	96.07M	14.41G	95.20M	14.04G	97.45%	93.94%	51.93%
