# Supplementary material for: Correlation of Coding and Non-Coding RNAs on the Fat Deposition of Yaks Under Different Feeding Systems
Source: Int J Mol Sci. 2025 Jun 3;26(11):5359. doi: 10.3390/ijms26115359 (PMC12154009; doi:10.3390/ijms26115359)
Supplement: Supplementary file 1 [file ijms-26-05359-s001.zip › Table S10 The composition of total mixed ration (TMR), and the common nutrition and content of main fatty acid in grass, TMR (air-dry basis)..pdf]

TABLE S10. The composition of total mixed ration (TMR), and the common nutrition and content of main fatty acid in grass, TMR (air-dry basis).

Item	TMR	Natural grass
Ingredient (%)		
Corn	19.20	-
Wheat bran	9.20	-
Whole corn silage	32.00	-
Oat Hay	28.00	-
Rapeseed meal	8.10	-
$NaHCO_3$	1.00	-
NaCl	1.50	-
Premix	1.00	-
Total	100.00	-
Common nutrition (%)		
Crud fat	4.52	2.63
Crud peotein	16.96	11.93
Neutral detergent fiber	23.24	76.14
Acid detergent fiber	13.84	10.09
Calcium	0.79	5.22
Phosphorus	0.37	0.07
Fatty acid (%)		
C16:0	0.68	0.30
C18:0	0.21	0.09
C18:1	0.18	0.07
C18:2n6	0.58	0.29
C18:3n3	1.42	0.68
